# Magnetically Driven Quadruped Soft Robot with Multimodal Motion for Targeted Drug Delivery

**DOI:** 10.3390/biomimetics9090559

**Published:** 2024-09-16

**Authors:** Huibin Liu, Xiangyu Teng, Zezheng Qiao, Wenguang Yang, Bentao Zou

**Affiliations:** 1 School of Electromechanical and Automotive Engineering, Yantai University, Yantai 264005, China; cactusil@163.com (H.L.); 15166828596@163.com (X.T.); qiaojingbiao6656@163.com (Z.Q.); 2 Engineering Training Center, Yantai University, Yantai 264005, China

**Keywords:** quadruped soft robot, magnetically driven, multimodal motion, targeted drug delivery

## Abstract

Untethered magnetic soft robots show great potential for biomedical and small-scale micromanipulation applications due to their high flexibility and ability to cause minimal damage. However, most current research on these robots focuses on marine and reptilian biomimicry, which limits their ability to move in unstructured environments. In this work, we design a quadruped soft robot with a magnetic top cover and a specific magnetization angle, drawing inspiration from the common locomotion patterns of quadrupeds in nature and integrating our unique actuation principle. It can crawl and tumble and, by adjusting the magnetic field parameters, it adapts its locomotion to environmental conditions, enabling it to cross obstacles and perform remote transportation and release of cargo.

## 1. Introduction

As medical standards advance and technology progresses, biomedicine is increasingly moving towards standardized procedures and targeted drug delivery [1,2]. In this context, soft robots exhibit significant potential due to their compact size, flexible manipulation, and programmable operation [3,4]. Because of their reduced size, soft robots are usually powered by external energy sources, such as magnetic, ultrasonic, electric, optical, or chemical fields [5,6,7,8,9]. Magnetic soft robots have been widely studied because the low-intensity magnetic fields used to drive them are harmless to biological cells and tissues and they offer programmable manipulation capabilities [2,10].

In terms of a single structure, current magnetic soft robots are mainly divided into two categories: continuum and untethered soft robots. Compared to continuum structures, untethered magnetic soft robots show higher flexibility in terms of maneuvering and workspace. Moreover, the untethered design allows for truly noninvasive treatments to a certain extent [2,11]. Zhao et al. proposed a magnetic robot kinematics mechanism based on magnetic moment-induced asymmetric friction effects, which enables a bipedal robot to maneuver flexibly at a speed of 25.33 BL/s fine in advance and to achieve a loading capacity of up to four times its own weight [12]. Du et al. designed a magnetic fish-shaped microrobot with a multifunctional tail, which is highly adaptable to avoid obstacles, as well as pass through a 45 μm wide channel, and morph and change colors in a complex environment with a height of 2 mm [13]. Hu et al. designed millimeter-scale magnetic microrobots with multiple motion modes that can roll, jump, swim, crawl, and transport goods two times their own weight under the manipulation of an externally programmed magnetic field [14]. Soon et al. designed a double-layer soft robot with programmable deformation inspired by the pangolin, which was able to achieve >70 °C heating at a distance of >5 cm in <30 s. It proved to be effective in cargo release, in situ demagnetization, thermotherapy, and hemostasis experiments on isolated tissues [15]. Although the aforementioned footless or biped designs present excellent multimodal locomotion in unstructured environments, developing a magnetically driven quadruped soft robot that walks like quadrupeds still faces many challenges [16,17]. In microrobotics, quadruped structures offer more stable support and locomotion for soft robots, enhancing both their operational accuracy and their ability to avoid obstacles and adapt to various environments [18,19]. Therefore, it is important to study the design of magnetic quadruped soft robots with strong locomotion and operational capabilities for their potential biomedical applications.

**Figure 1:**
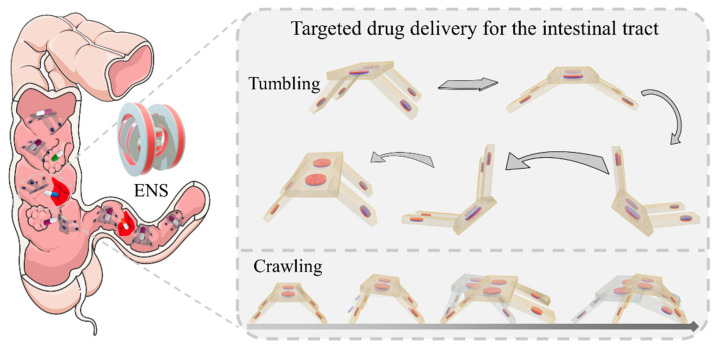
Schematic representation of the two motion modes and targeted drug delivery of a magnetically driven quadruped soft robot.

In this work, we present a bionic magnetic quadruped soft robot for intestinal-targeted drug delivery (Figure 1). This robot consists of a magnetic top cover and four flexible feet, each with specific magnetization directions. Its magnetic responsiveness is derived from N52 NdFeB magnetic particles, and it possesses two modes of motion, crawling and tumbling, under the manipulation of a programmed magnetic field. Moreover, the combination of these two motion modes enables the transportation and release of cargo to a specified location. In addition, we analyzed the motion principle and motion model of the quadruped structure and verified the motion characteristics of its two motion modes, its ability to cross obstacles, and its ability to transport and release cargo through experiments. The theoretical analysis and experimental results show that our magnetically driven quadruped soft robot has multi-modal motion capability, which provides a typical case for the design and research of magnetic quadruped microrobots. Moreover, the excellent motion characteristics and manipulation operation capability are expected to be applied to targeted drug delivery.

## 2. Material and Methods

### 2.1. Design and Fabrication of Magnetically Driven Quadruped Soft Robot

**Figure 2:**
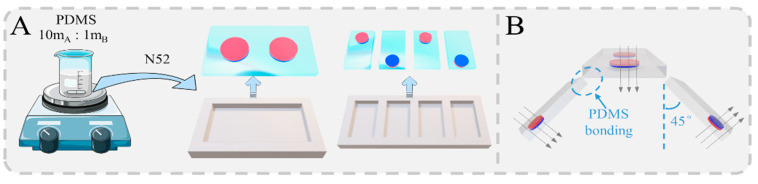
Preparation and assembly of magnetic quadruped soft robot. (**A**) Preparation of magnetic quadruped soft robot. (**B**) Assembly of magnetic quadruped soft robot.

Inspired by the way crabs move in nature, we designed a magnetically driven quadruped soft robot that mimics the movement of crabs. This robot consists of a magnetic top cover and four feet, each with specific magnetization directions. Both the top cover and the feet are composed of polydimethylsiloxane (PDMS) and N52 NdFeB magnetic particles. First, the PDMS colloid was formulated according to the ratio (A:B: 10:1), and the molds of the top cover and the feet were prepared using a high-precision 3D printer (Figure 2A). The A and B formulations of PDMS before mixing and curing are clear fluids. After homogeneous mixing, the PDMS prepolymer remains a clear solution with a viscosity of 4000 at 25 °C room temperature. Next, the N52 magnetic particles were placed in the designated positions in the mold and poured into the formulated PDMS colloid for heating and curing. Modally stable PDMS flakes were obtained after curing at 80 °C ambient for 1 h or at 100 °C ambient for 30 min. The position height of the magnetic pellet was slightly adjusted during the curing process so that it was completely encapsulated by the PDMS. At this stage, the sheet exhibited a hardness of 50 Shore A, a tensile strength of 7 MPa, and an elongation at break of 140%. After curing and cooling to room temperature, the flexible hydrogel of the top cover and foot were carefully detached from the mold. According to the designed dimensions of the mold, the magnetic top cover was prepared with a length of 18 mm, a width of 8 mm, and a thickness of 0.9 mm. The four magnetic feet were prepared with the same dimensions, specifically with a length of 11 mm, a width of 4 mm, and a thickness of 0.9 mm. In addition, the N52 magnetic particles for the top cover were cylindrical, with a diameter of 4 mm and a thickness of 0.5 mm. For the feet, the N52 magnetic particles were also cylindrical but with a diameter of 2 mm and a thickness of 0.5 mm. As shown in Figure 2B, the four flexible feet were arranged with a specific magnetization direction, ensuring that each foot was positioned at a 45° angle relative to the top cover and the magnetic particles were oriented in different directions. The feet were then bonded to the top cover using PDMS.

### 2.2. Magnetic Field Properties and Magnetic Drive Bending Model

**Figure 3:**
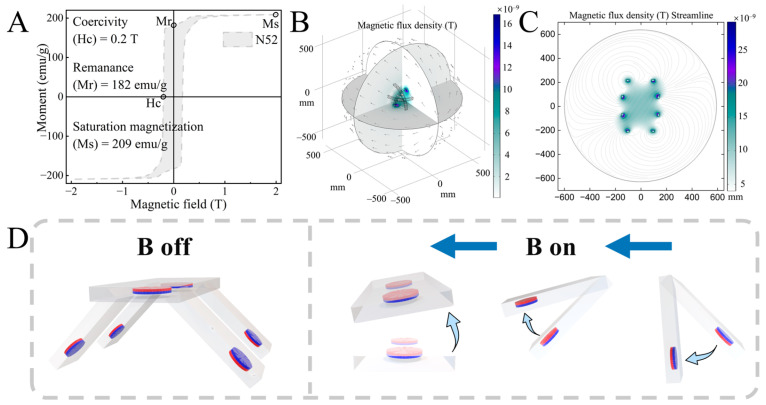
Material parameters and magnetic field properties and magnetically actuated deformation of magnetic quadruped soft robot. (**A**) Material parameters of N52 NdFeB magnetic particles. (**B**) Simulation of ENS in COMSOL with multi-cut magnetic field distribution. (**C**) Simulation of ENS in COMSOL with magnetic field distribution in the work plane. (**D**) Deformation effect of the robot in response to ENS actuation.

Compared to NdFeB and Fe_3_O_4_ magnetic powders, N52 NdFeB magnetic particles have a stronger magnetic field response. The magnetic particles used in this study were purchased from Shenzhen Lala Magnetic Materials Development Co., Ltd., Shenzhen, China. They were measured using a vibrating sample magnetometer (VSM) and the obtained material data were processed, as shown in Figure 3A. The N52 grains we used have a remanent magnetization (Mr) of 182 emu/g and a coercivity (Ms) of 209 emu/g. The grains have excellent remanent magnetization and coercivity, enabling them to generate a strong driving torque in response to manipulation by a uniformly strong magnetic field. In addition, we used a programmable and highly maneuverable 3D Helmholtz coil electromagnetic field as an electromagnetic navigation system (ENS). In the uniformly strong field generated by the electromagnetic coil, the deformation of the magnetic top cover and foot was driven by torque to achieve ordered motion. Figure 3B,C illustrate the magnetic field distribution during the operation of the ENS. The simulations across multiple sections and work planes reveal that the magnetic field is uniformly distributed in the three-dimensional operation space along a certain direction. Moreover, by adjusting the current signal of the coil, it is possible to establish a uniform magnetic field in any direction in space.

The specifically assembled robot exhibits different deformations of the joints in each magnetization direction in response to manipulation by the ENS. However, since the two legs on the same side have the same magnetization direction, their deformations when facing the same homogeneous magnetic field are also the same. As shown in Figure 3D, when the ENS is off, the assembled robot relies on the support of the quadruped to maintain a smooth attitude. When the ENS is turned on and provides a uniformly strong field to the left, the top cover, the left two feet, and the right two feet deform as shown in Figure 3D. This deformation results from the coupling of the magnetic moment and the deformation between the constituent structures. Based on such ordered deformation characteristics, we can program the direction and magnitude of the magnetic field in the ENS to manipulate the motion of the robot.

### 2.3. Two Motion Modes for Magnetically Driven Quadruped Soft Robot

**Figure 4:**
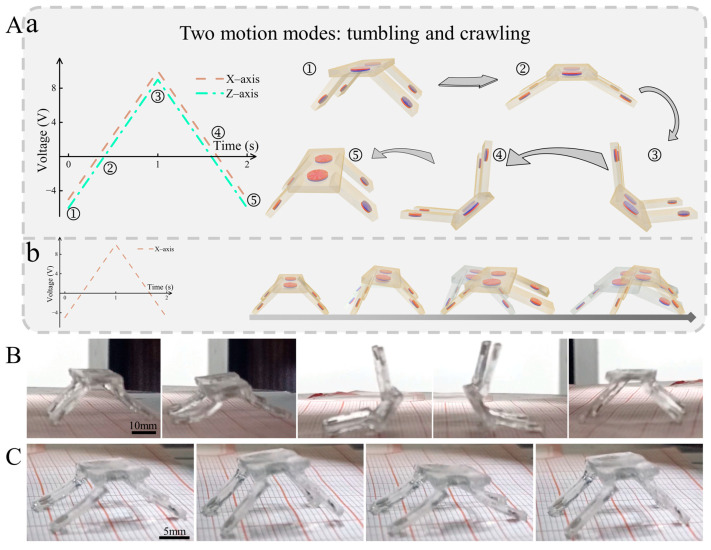
Manipulation of two motion modes. (**A**) Manipulation signal and motion decomposition diagrams for two motion modes, (a) tumbling and (b) crawling. (**B**) Experimental screenshot of tumbling motion. (**C**) Experimental screenshot of crawling motion.

Here, we manipulated the robot using two different signals of the drive field to realize two motion modes, tumbling and crawling. As shown in Figure 4A(a), the current signal for manipulating the robot’s tumbling motion consists of triangular waves on the *X*-axis and *Z*-axis. Through superposition, a uniform magnetic field of varying size and direction is generated in the ENS operation space. The tumbling motion occurring in the robot driven by this uniform field can be decomposed into five deformation steps as described in ①–⑤. The deformation steps correspond to the signal diagrams. First, in the ①–② process, the magnetic field in the XZ plane, angled diagonally downward, causes the left and right quadrupeds to extend outward, and the robot’s overall attitude is downward. Then, in the ②–③ process, the magnetic field direction is reversed and gradually intensified, causing the robot to undergo tumbling deformation. Next, in the ③–④ process, the magnetic moment gradually decreases and, in the coupling with gravity, the top cover of the robot gradually makes contact with the support plane. Finally, in the ④–⑤ process, the magnetic field direction is again reversed and gradually intensified, completing the tumbling deformation and restoring the initial attitude under the dual response of the feet and the top cover. Figure 4B illustrates the deformation process of the robot, showing how it undergoes tumbling motion as manipulated by this signal in the experiment. Based on different motion postures, the manipulated deformation of the tumbling motion is realized by adjusting the size and direction of the magnetic field in the operation space. Moreover, the observed physical deformations are consistent with our theoretical analysis that the tumbling process of magnetic quadruped microrobots includes four steps of attitude downward pressure, lateral tumbling, and back to the correct attitude. In addition, subsequent experiments verified that its motion characteristics can be adjusted by changing the magnetic field strength and frequency. In addition to tumbling motion, the robot can also be manipulated to perform a crawling motion by adjusting the drive signal. As shown in Figure 4A(b), a single *X*-axis drive signal drives the robot to mimic the crawling motion of a crab. As with the tumbling motion, the top cover and quadruped are magnetically bent and deformed during crawling. It should be noted that the magnetic field strength is reduced by adjusting the size of the input current, so that the top cover responds with less deformation, which is insufficient to induce a tumbling motion. At this time, the top cover exhibits the deformation characteristic of lifting up at a small angle under the drive of the ENS. Additionally, coupled with the bending deformation of the four feet, different deformations of the left and right feet are realized by decreasing or increasing the support force of the feet. By combining these motion features and programming the control signals, a crab-like crawling motion is realized. First, the left side of the top cover is lifted to promote the contraction of the left two feet while inhibiting the movement of the right two feet. Then, the magnetic field is reversed and the right side of the top cover is lifted to promote the extension of the right two feet and inhibit the movement of the left two feet. By repeating the above deformation processes and combining them, an effective movement similar to crab crawling is realized. Again, we verified this analysis in our experiments. As shown in Figure 4C, the deformation effects of the feet and the top cover during the crawling motion in the experiment are basically consistent with our analysis. Based on different motion postures, the manipulation of the crawling motion is realized by adjusting the size and direction of the magnetic field in the operation space. And the observed physical deformation is consistent with our theoretical analysis that the magnetic quadruped microrobots’ crawling process includes four steps of posture down, alternating unilateral warping, and returning to the correct posture. We also explored how variations in magnetic field strength and frequency affect the crawling motion characteristics in subsequent experiments.

## 3. Results and Discussion

### 3.1. Deformation Response of the Magnetically Driven Quadruped Soft Robot

**Figure 5:**
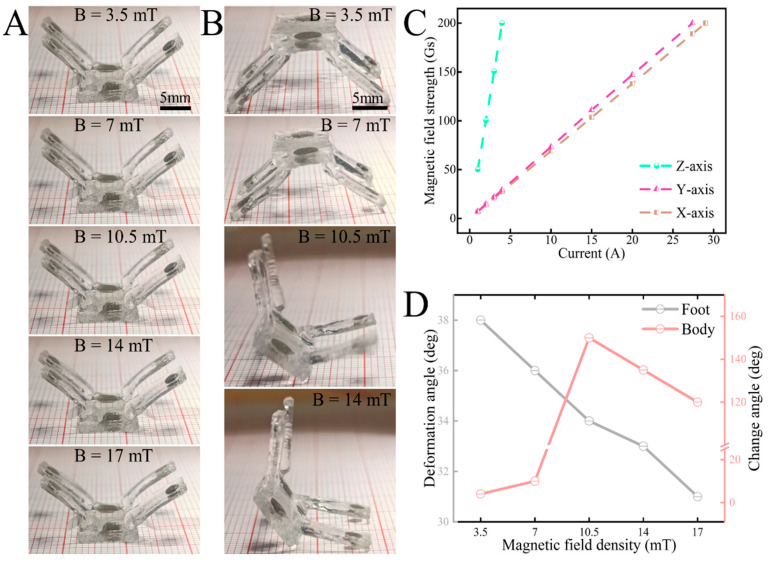
Deformation response of the magnetically driven quadruped soft robot. (**A**) Bending response of the robot’s feet. (**B**) Response of the robot to tumbling deformation. (**C**) Conversion of magnetic field input current of solenoid coil versus magnetic field strength. (**D**) Driving effect of magnetic field strength on foot bending and top cover deformation.

The effect of magnetic field strength on the bending deformation of the robot was investigated. As shown in Figure 5A, the strength of the external DC magnetic field gradually increases while the robot is flipped and fixed on a flat surface. We also observed and counted the bending deformation angle of the foot. The deformation angle of the foot in the fixed attitude gradually increases with the gradual increase in the external magnetic field strength. The quadruped deformation angle of the robot gradually increases from the initial 45° to 55°. Moreover, the robot quadruped deformation angle is positively correlated with the magnetic field strength. It should be noted that the robot in the natural attitude may change its attitude due to the coupled magnetic response of the magnetic top cover, which, in turn, affects the deformation angle of the foot. In addition, to investigate the effect of magnetic field strength on the attitude of the robot, we applied a gradually increasing magnetic field to the robot in its natural position. We then observed and counted the robot’s attitude as well as the deformation angle of the top cover at each magnetic field strength. As shown in Figure 5B, the extension inclination angle of the quadruped gradually increases with higher magnetic field strength, resulting in a downward compression of its attitude. Moreover, the magnetic top cover responds to the magnetic field drive and deforms under the action of torque to produce a certain inclination angle. With the increase in the inclination angle, the tumbling motion characteristic eventually appears. Furthermore, due to the coupling effect of the robot’s gravity and magnetic moment, the magnetic top cover establishes a specific angular relationship with the support plane. As the magnetic moment increases, the coupled attitude aligns more closely with the direction of the magnetic field. In general, the motion attitude of magnetic quadruped microrobots is positively correlated as a function of magnetic field strength. And the magnetic quadruped microrobots’ motion characteristics and attitude can be controlled by adjusting the magnetic field strength and direction. The magnetic field of the ENS is generated by an electromagnetic coil, and its strength can be controlled by adjusting the input current. The relationship between the input current and the magnetic field strength of the 3D Helmholtz coil triaxial is illustrated in Figure 5C. By adjusting the size and direction of the triaxial magnetic field in space, a uniform magnetic field of any desired direction and size can be synthesized. In addition, the statistical data on how the magnetic field strength affects the robot were organized and are presented in Figure 5D. From the intersection of the data in the figure, it can be seen that the robot used in the experiment tumbled and deformed, i.e., changed its attitude, at a magnetic field strength of 11.5 mT. At this strength, the magnetic field’s impact on the quadruped’s deformation also changes. Moreover, after the experimental test, it can be obtained that, under the action of the field strength at this transition point, 9 motion modal transitions occurred in 10 motion controls, and the stability is as high as 90%. Recognizing this will aid in analyzing the control and transformation between the robot’s two motion modes: crawling and tumbling. This will also help us further analyze how the magnetic field frequency affects the motion speed of the two motion modes in the corresponding magnetic field strength interval.

Since the motion of magnetic quadruped microrobots is mainly obtained by deforming the PDMS sheet wrapped with N52 magnetic particles, it is necessary to examine the deformation effect of the PDMS sheet under various ambient temperatures. Accordingly, only if the PDMS sheet maintains good deformation performance, the designed programmed motion can be realized. The material specification shows that the cured PDMS maintains stable mechanical properties in the temperature interval of −50 to 200 °C. In addition, its thermal conductivity is 0.17 W/(m‧K), which has a certain temperature resistance and can better meet the operational requirements. Combined with the characteristics of the operating environment, the PDMS sheet still maintains good elasticity and deformation ability under the environment of −4 °C after experimental testing. Moreover, since the PDMS sheet is cured by heating, it maintains stable mechanical properties even under a high-temperature environment. Therefore, the PDMS sheet for responding to magnetic torque deformation has good operational stability at various ambient temperatures.

### 3.2. Kinematic Characterization of the Magnetically Driven Quadruped Soft Robot

**Figure 6:**
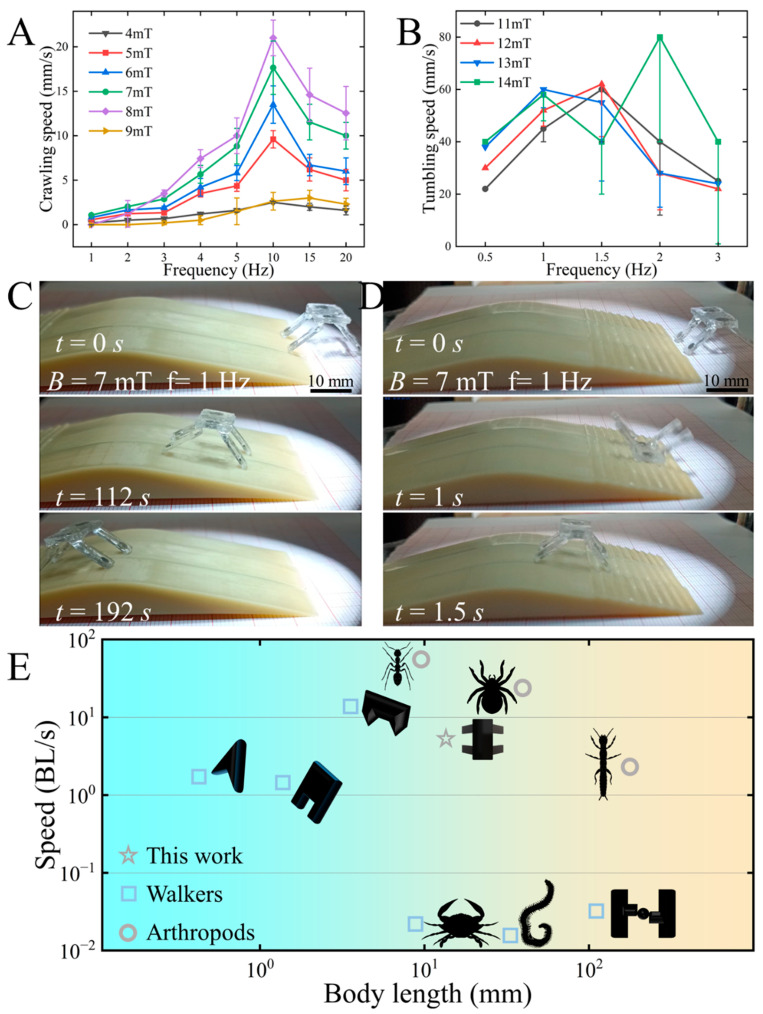
Kinematic characteristics of magnetically driven quadruped soft robot and its ability to traverse obstacles. (**A**) Effect of magnetic field strength and frequency on the robot’s crawling kinematic speed. (**B**) Effect of magnetic field strength and frequency on the robot’s tumbling kinematic speed. (**C**) The robot crawling through an obstacle. (**D**) The robot tumbling through an obstacle. (**E**) Performance comparison with reported robots.

Previous analyses and experiments indicate that the magnetic quadruped soft robot may experience tumbling attitude changes under a magnetic field strength of 11 mT. Therefore, in this section, we set the maximum magnetic field strength for crawling at 9 mT and the minimum for tumbling at 11 mT. We then further investigated the motion characteristics of these two motion modes by adjusting the magnetic field frequency and strength within the intervals. As shown in Figure 6A, we statistically analyzed how the magnetic field frequency affects the motion speed of the magnetic quadruped soft robot within a magnetic field strength range of 4 mT to 9 mT. The figure shows that the magnetic quadruped soft robot exhibits lower motion velocity at all frequencies when the magnetic field strength is 9 mT. Observations during the experimental process reveal that this is due to the increased deformation angle of the magnetic top cover at larger magnetic field strengths. This greater deformation significantly reduces the stability and effectiveness of the robot’s motion and can even cause unexpected deformation during tumbling. The robot also moves at the same lower speed at all frequencies when subjected to a 4 mT magnetic field strength. However, the difference is that the smaller magnetic field strength results in a smaller deformation angle of the magnetic top cover, which limits the bending deformation of the foot and thus leads to a lower movement speed. The motion characteristics of the robot at each frequency under a magnetic field strength of 5 mT to 8 mT are also affected by the deformation angle of the magnetic top cover and the degree of foot bending deformation. Overall, the robot exhibits better motion speeds when subjected to 7 mT and 8 mT magnetic field strengths. However, at 8 mT, the robot may tumble and deform when driven in a lower-frequency magnetic field, which can prevent it from completing the crawling motion. Through observation and analysis of the experimental phenomena, we have identified a compelling reason: at 8 mT, the magnetic top cover exhibits a larger deformation angle, increasing the likelihood of tumbling deformation. Under high-frequency magnetic fields, the timely reversal of moments prevents tumbling deformation caused by inertia. Conversely, at low magnetic field frequencies, tumbling deformation occurs due to inertia. Therefore, for the purpose of crawling motion drive in this study, a magnetic field strength of 7 mT is more appropriate.

In general, as the magnetic field frequency increases, the motion cycle time decreases, resulting in faster speeds. However, observing the robot speed versus frequency curve in the figure reveals that, as the frequency increases, the motion speed first increases and then appears to decrease. Observations during the experiment indicate that, when the frequency increases to a certain degree, the deformation and recovery speed of the robot cannot keep pace with changes in the magnetic field frequency. Therefore, the stability and effectiveness of the motion decrease, leading to a natural reduction in speed. For this experimental phenomenon, we adopt the definition from other studies and refer to it as the cutoff frequency, i.e., the maximum frequency at which the robot can match changes in the magnetic field. Clearly, the cutoff frequency for the robot’s crawling motion in our study is 10 Hz. Through experimental analysis, we can further optimize the motion effect by choosing the appropriate magnetic field strength and frequency within the parameter interval of the crawling motion mode according to the operational requirements.

In addition, we conducted a statistical analysis of how the magnetic field frequency affects the robot’s tumbling motion mode within varying intervals of magnetic field strength. As shown in Figure 6B, we tested the tumbling motion speed of the robot at each magnetic field frequency within the magnetic field strength varying from 11 mT to 14 mT. It can be seen that the motion speed of the robot increases gradually with the increase in the magnetic field strength. However, when the magnetic field strength reaches a certain degree, the motion speed of the robot shows little increase and even decreases at certain frequencies. Based on the experimental process, we found that the deformation of the robot’s feet also increases as the magnetic field strength increases. However, as the bending of the foot increases, the bending moment increases dramatically, leading to a dramatic increase in the required magnetic moment, which, in turn, requires a stronger magnetic field strength. At this stage, the regular incremental increase in magnetic field strength is insufficient to provide the required magnetic moment. 

The robot’s speed is also affected by the magnetic field frequency, and we tested its movement speed across a frequency range of 0.5 to 3 Hz. As illustrated in the figure, the tumbling motion speed gradually increases as the magnetic field frequency increases. However, as the magnetic field frequency continues to increase, the speed of the tumbling motion starts to decrease. During the tumbling motion experiment, we observed a cutoff frequency phenomenon similar to that seen in the crawling motion. Additionally, due to the larger deformation involved in the tumbling motion, it is more sensitive to changes in the magnetic field frequency. According to the experimental data, the tumbling motions all showed a certain speed decrease at a magnetic field frequency of 1.5 Hz, with the speed continuing to decrease as the frequency increased. However, we also noticed that the robot reached a tumbling motion speed of 80 mm/s at 2 Hz under a magnetic field strength of 14 mT. Several experiments have confirmed that the robot does not exhibit a low tumbling motion speed. We found that, while higher magnetic field strength can cause instability in the tumbling motion, increasing the magnetic field frequency makes the acting time of the magnetic moment shorter. Instead of optimizing this problem, this results in an abnormal increase in speed.

Through an in-depth investigation of the motion characteristics of the robot, we identified and summarized the characteristic parameters of effective motion. There is a situation where different parameters can achieve the same control effect for these motion control parameters. For this situation, we take improving the stability of motion manipulation and reducing the accidental damage to human organs and tissues as the guideline and choose as much as possible a low magnetic field frequency (improve the stability of the motion) and a small magnetic field strength (avoid the unknown damage under the high field strength). Moreover, according to the operational requirements, we flexibly adjust the parameters of the control variables so as to achieve the best control effect. Building on these parameters, we conducted an experimental test to investigate how adjusting the robot’s manipulation parameters according to its motion characteristics can enhance its ability to navigate through obstacles. The complete experimental procedure is detailed in Video S1, and Figure 6C,D provide representative screenshots of the experiments. As shown in Figure 6C, the robot can pass through a bridge-shaped environment with a certain slope using a crawling motion. When facing a sloped environment, its motion capability can be enhanced by adjusting the inclination angle of its crawling motion, based on the crawling motion characteristics analyzed earlier, so as to navigate the slope smoothly. However, while it can navigate a slope through crawling, it takes a considerable amount of time. Therefore, further exploration and optimization are needed to maintain a stable attitude and movement speed. On the other hand, the robot can traverse a ramp with a cross-grain in 1.5 s and return to its initial upright attitude (Figure 6D). In this case, the robot’s locomotion capability and speed are greatly improved. However, the tumbling process implies a drastic change in attitude, which can negatively impact cargo stability during transport. Therefore, it is important to find a balance between maintaining cargo stability and achieving movement speed in the tumbling motion mode. As the magnetic quadruped microrobots can flexibly change the two motion modes of crawling and tumbling, therefore, the motion characteristics can be flexibly adjusted according to the operational requirements during the actual operation so as to meet the positioning accuracy and speed requirements.

After experimental testing and analytical verification, our designed magnetic quadruped microrobots have two freely switchable motion modes and excellent motion characteristics. In order to further highlight the advantages of the motion characteristics, we made a comparison with some typical studies that have been reported. We selected some typical walking untethered magnetic microrobots as well as the locomotion characteristics of arthropods in nature for comparison. The comparisons were made in terms of body size and locomotion speed, respectively, to visualize the advantages and characteristics of each magnetically driven microrobot design. For the data to be more informative, we use the body length and the number of moving body lengths per second as comparison parameters. As can be seen from the data in Figure 6E, the magnetic microrobots that currently exist include multiple orders of magnitude sizes ranging from 0.1 mm to 100 mm. Moreover, they have different motion characteristics based on design features, for example, triangular [20], portal [21], biconical [12], multipedal [22], crab [23], and bionic crab shapes [24] with walking ability. Among them, the magnetic quadruped bionic microrobots we designed have excellent locomotion characteristics by achieving the first gradient of locomotion speed while maintaining the more common body size. On the other hand, comparing with typical arthropods in nature, such as ants, spiders, web spinners, Hediste diadroma, etc., the magnetic quadrupedal microrobots still have comparable locomotion speeds and locomotion characteristics.

### 3.3. Transportation and Release of Cargo by the Magnetically Driven Quadruped Soft Robot

**Figure 7:**
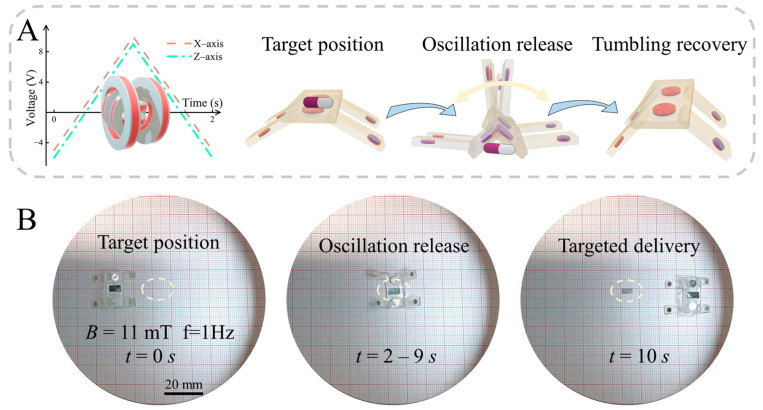
The magnetic quadruped soft robot transporting and releasing cargo. (**A**) Schematic diagram of the robot transporting and releasing cargo using tumbling and swinging motions. (**B**) Screenshots of experiments of the robot transporting and releasing cargo using tumbling and swinging motions.

In the previous section, we explored and verified the kinematic properties and the obstacle-crossing ability of the designed robot. Based on this research and experimental observations, we designed in vitro simulated cargo transportation experiments. Considering the characteristics of the two motion modes, crawling and tumbling can be combined to effectively navigate a complex operating environment. First, the magnetic quadruped microrobots are maneuvered to the target location. Then, the magnetic quadrupedal microrobots’ tumbling motion characteristics are utilized to adjust the control signal so that they tumble rapidly and continuously at the targeted drug delivery position to achieve drug release. After realizing the targeted drug delivery operation, the magnetic quadruped microrobots’ tumbling motion control signal is restored. The magnetic quadruped microrobots are made to complete the tumbling motion, recover the attitude, and leave the operation area. As shown in Figure 7A, by adjusting the control signal of the electromagnetic coil and the magnetic field strength, the characteristic attitude of the tumbling motion can be changed, thus realizing the half-tumbling motion with left and right swaying. Once the cargo has been released, restoring the magnetic field parameters of the tumbling motion will enable the soft robot to recover its attitude. Based on this control strategy, we designed and carried out experiments for cargo transportation and release. The complete experimental procedure can be seen in Video S2, with representative screenshots provided in Figure 7B. As shown in Figure 7B, after crawling and tumbling to reach the target position, the robot performs a half-tumbling motion in response to the control signal of the tumbling motion (Target position). Then, the magnetic field strength and frequency are varied to perform a left–right swinging motion to release the transported cargo (Oscillation release). Finally, the tumbling motion is resumed to control the magnetic field, thus adjusting the robot’s attitude to upright (Targeted delivery). This process combines the stability and the ability of the crawling motion to handle small displacements with the ability of the tumbling motion to manage large displacements and release cargo through oscillation. Based on this mode of operation, the ability to achieve work accuracy and movement speed is greatly enhanced. From the experimental observations, we successfully applied the magnetic quadruped soft robot for cargo transportation and release following the designed experimental steps. However, several areas require improvement, such as enhancing the stability during cargo transportation and addressing challenges related to cargo release. In the future, our goals include further reducing the robot’s size, enhancing its movement ability based on its kinematic characteristics, and optimizing the methods for cargo transportation and release.

## 4. Conclusions

In this work, we have introduced a bionic magnetic quadruped soft robot featuring a magnetic top cover and four magnetic flexible feet with specific magnetization angles. We introduced and analyzed the material parameters and magnetic field properties of the robot, including the hysteresis loop of magnetic particles and the deformation of magnetic flexible feet under magnetic field drive. After experimental testing, the overall structure maintains stable mechanical deformation properties in environments ranging from −4 °C to 80 °C. Then, we analyzed and verified through experiments two motion modes of the robot, crawling and tumbling, controlled by magnetic field parameters. In addition, we analyzed the effect of magnetic field strength on the motion modes of crawling and tumbling, considering their respective attitude characteristics. By adjusting the parameters of the control variables, the designed magnetic quadruped microrobots are able to achieve a motion speed of 80 mm/s. According to the experimentally obtained magnetic field strength interval, the stability of modal transition is as high as 90% and we further explore the motion characteristics of the two motion modes. Finally, we experimentally verified the robot’s robust ability to traverse obstacles and transport and release cargo in a targeted manner and also demonstrated its effectiveness and promising application for tissue-targeted drug delivery in tissues such as the intestinal tract. Considering the operational requirements and issues identified in this study, our next step will be to further reduce the size of the robot, e.g., by preparing a magnetic microrobot body using an optical 3D printing system based on digital micromirror devices (DMD). Ferromagnetic compound particles are used instead of N52 magnetic particles, resulting in more flexible setting of the magnetization direction as well as shrinking the volume of the magnetic response layer. In addition, the structure design is further optimized to enhance the motion characteristics. Finally, according to the operational requirements, we try to increase the operational module child to optimize the operational process and enhance the application potential.

## Data Availability

The data that support the findings of this study are available on request from the corresponding author.

## Supplementary Materials

The following are available online at https://www.mdpi.com/article/10.3390/biomimetics9090559/s1, Video S1: The robot crawling and tumbling through an obstacle. Video S2: The robot transporting and releasing cargo using tumbling and swinging motions.
